# Assessing functional recovery shortly after knee or hip arthroplasty: a comparison of the clinimetric properties of four tools

**DOI:** 10.1186/s12891-016-1338-7

**Published:** 2016-11-16

**Authors:** Stéphane Poitras, Kristi S. Wood, Jacinthe Savard, Geoffrey F. Dervin, Paul E. Beaulé

**Affiliations:** 1 School of Rehabilitation Sciences, University of Ottawa, 451 Smyth Road, Ottawa, ON K1H 8M5 Canada; 2 Department of Orthopaedic Surgery, The Ottawa Hospital, 501 Smyth Rd, Ottawa, ON K1H 8L6 Canada

**Keywords:** Arthroplasty, Outcomes assessment, Function

## Abstract

**Background:**

Following hip or knee arthroplasty, it is clinically warranted to get patients functional as quickly as possible. However, valid tools to assess function shortly after knee or hip arthroplasty are lacking. The objective was to compare the clinimetric properties of four instruments to assess function shortly after arthroplasty.

**Methods:**

One hundred eight patients undergoing hip or knee arthroplasty were assessed preoperatively, 1 and 2 days postoperatively, and 2 and 6 weeks postoperatively with the Timed Up and Go (TUG), Iowa Level of Assistance Scale (ILAS), Postoperative Quality of Recovery Scale (PQRS), and Readiness for Hospital Discharge Scale (RHDS). Descriptive data, floor and ceiling effects, responsiveness, interpretation and construct validity were determined.

**Results:**

Only the ILAS and RHDS support subscale demonstrated floor or ceiling effects. A large deterioration from preoperative to postoperative, followed by large improvements after surgery were seen in the TUG and ILAS scores. The RHDS personal status subscale and the PQRS pain and function dimensions demonstrated large improvements after surgery. Changes in the RHDS global scale and personal status subscale, PQRS pain dimension and TUG were significantly related to patient perceived improvement. Minimal important changes were obtained for the RHDS global (1.1/10) and personal status subscale (2.3/10), and the TUG (43.4 s at 6 weeks). For construct validity, the PQRS function dimension and RHDS were moderately related to the TUG or ILAS. The correlation between TUG and ILAS was high from preoperative to postoperative day 2, but substantially decreased at 2 and 6 weeks.

**Conclusions:**

The TUG and RHDS personal status subscale demonstrated the best clinimetric properties to assess function in the first 6 weeks after hip or knee arthroplasty.

## Background

Following hip or knee arthroplasty, it is clinically warranted to get patients functional as quickly as possible. This reduces hospitalisation time, prevents deconditioning, increases patient safety and decreases the need for external resources after hospital discharge [1]. However, determining patient functional capacities after surgery requires a valid outcome measure. Having a valid short-term function outcome measure after arthroplasty also allows to evaluate the efficacy of perioperative interventions aimed at improving function shortly after surgery, such as preoperative management, surgery/anesthesia protocols and postoperative management during hospitalization. Although several outcomes measures have been demonstrated as valid to assess function following hip or knee arthroplasty, such as the Lower-Extremity Functional Scale (LEFS), Western Ontario & McMaster Universities Osteoarthritis Index (WOMAC), Knee injury and Osteoarthritis Outcome Score (KOOS)/Hip disability and Osteoarthritis Outcome Score (HOOS) and Oxford Knee Score, they have been validated for long-term function [2]. These instruments have also shown validity problems when assessing function shortly after surgery, notably because they assess more advanced function such as squatting, kneeling, jumping, running and heavy domestic duties [3–5]. They also have been developed for the symptoms of osteoarthritis, not specifically for functional recovery shortly after surgery.

To assess function, there are two broad category of tools: patient-reported outcome measures (PROMs) and performance outcome measures [6]. With PROMs, the patient self-reports their perceived function through questionnaires. With performance measures, the patient actually performs one or several functional tasks and is scored on the capacity to perform the task. Following an overview of the literature, promising and most used PROMs and performance measures were identified in order to compare their clinimetric properties to assess function shortly after hip or knee arthroplasty. This selection was based on the theoretical relevance of tools to assess functional capacities after knee or hip arthroplasty (face validity), their frequency of clinical use in patients undergoing arthroplasty [7], or promising results in previous studies.

For performance measures, the Timed Up and Go (TUG) and the Iowa Level of Assistance Scale (ILAS) were selected. The TUG assesses the time that a patient takes to rise from a chair, walk three metres, turn around, walk back to the chair, and sit down [8]. It has been demonstrated to predict both short [9] and long-term function [10, 11] following arthroplasty. The ILAS assesses the capacity of the patient to perform five tasks (supine to sitting, sitting to standing, walking, stairs, and walking speed), with a global score out of 50. It also takes into account the assistive devices needed to complete the tasks [12]. Both these tools are considered important performance assessments in patients with hip or knee osteoarthritis [6].

For PROMs, the Postoperative Quality of Recovery Scale (PQRS) and the Readiness for Hospital Discharge Scale (RHDS) were selected. Both of these tools have been specifically developed to assess recovery following surgery. The PQRS assesses the patient’s perceived status on four dimensions (pain, emotion, function, and cognition), with scoring determined by return to preoperative status [13]. The PQRS has shown good clinimetric properties in surgery patients other than arthroplasty [14], but has been used to study recovery patterns of patients undergoing knee arthroplasty [15]. The RHDS assesses the patient’s perceived readiness to hospital discharge on four dimensions: personal status, knowledge about what to do after discharge, coping ability and expected support [16]. The RHDS has been validated with various surgery populations other than arthroplasty [16, 17].

The objective of this study was to compare the clinimetric properties of these four instruments (TUG, ILAS, PQRS and RHDS) to assess functional recovery shortly after hip or knee arthroplasty.

## Methods

### Patient recruitment and data collection

Patients scheduled for primary unilateral partial or total knee or hip arthroplasty because of osteoarthritis in a university-affiliated hospital in Ottawa, Canada, were invited to participate to the study between March and October 2013. Patients were excluded if they had any of the following characteristics: knee or hip arthroplasty in the month preceding surgery; revision arthroplasty; diagnosed neurologic or musculoskeletal disease (excluding osteoarthritis) adversely affecting gait or weight-bearing; unable to read and/or understand English; documented cognitive impairment precluding questionnaire completion; under 18 years of age; not living in the area of the surgery hospital. Although there are no definitive criteria to determine sample size in clinimetric studies, it is recommend to assess at least 50 patients [18]. Thus, 54 patients undergoing knee arthroplasty and 54 patients undergoing hip arthroplasty were recruited, to take into account potential attrition. Ethical approval was obtained from the Ottawa Hospital ethical review board. All patients gave their informed written consent for participation in the study.

The TUG, ILAS and PQRS were assessed preoperatively, 1 and 2 days postoperatively, and 2 and 6 weeks postoperatively. The RHDS was assessed 1 and 2 days postoperatively, since it is not relevant preoperatively nor after hospital discharge. The TUG and ILAS were completed by trained physical therapists, the PQRS was done through interviews completed by trained research assistants, and the RHDS was self-completed by the patient. If a patient was not able to complete an assessment, reasons were noted by the assessor using a standardized chart. In order to reduce the influence of performance on patient perceptions, PROMs were completed before performance measures at each period. Demographic variables were also collected before surgery.

### Data analyses

The clinimetric properties studied follow the COnsensus-based Standards for the selection of health Measurement Instruments (COSMIN) statement [19]. The following properties were assessed: descriptive data; floor and ceiling effects; responsiveness; interpretation; and construct validity. Reliability was not assessed in this study for different reasons: 1) reliability for the TUG and ILAS has been previously demonstrated in this population [20–24]; 2) assessing reliability requires a stable patient condition, which is difficult to obtain for the RHDS since it is assessed during hospitalisation where the patient condition changes quickly. Tools demonstrated floor or ceiling effects if more than 15% of patients had the lowest or highest scores respectively at a time point [18]. Since the PQRS is a dichotomous outcome, floor and ceiling effects were not assessed but the proportion of patients deemed recovered according to the PQRS (return to preoperative status) was assessed at each period. To assess responsiveness, standardised response means (SRM) were calculated between two consecutive periods. The following thresholds were used for SRM interpretation: ‘trivial’ (under 0.20), ‘small’ (0.20–0.50), ‘moderate’ (0.50–0.80), or ‘large’ (>0.80) [25]. Since a SRM cannot be calculated for the PQRS because it is dichotomous, the proportion of patients evolving from “not recovered” to “recovered” between consecutive periods was calculated. For interpretation, the minimal important change (MIC) was calculated using an anchor-based method [26]. To determine the anchors, the following question was asked at each time period after surgery: “*How do you feel that your overall physical condition has changed since you woke up from surgery?”.* This was answered using a 5-point Likert scale ranging from “much worse” to “much better”. A patient was in the “improvement” category if the answer to the question improved from the postoperative day 1 answer. Receiver Operating Characteristics (ROC) curves were then produced to study the relationship between perceived improvement and score changes between periods, and identify an MIC if there was a significant relationship [19]. For the PQRS, Fisher’s exact test was used to study its relationship with the anchors and sensitivity/specificity was calculated if significant [19]. For construct validity, the relationship between tools was assessed with either Pearson correlation coefficients or Student t-tests depending on the nature of the variables, where it was hypothesized that the relationship between performance measures would be strong, and the relationship between PROMs and performance measures would be weak. Missing variables of the PROMs were addressed using the respective authors’ instructions. Patients unable to perform the TUG were given a surrogate time, corresponding to the slowest time of the entire study for all subjects [27]. There were no missing values for the ILAS. Analyses were performed separately for knee and hip arthroplasty. All statistical analyses were performed using SPSS version 21 (SPSS Inc, Chicago, Illinois). A two-tailed level of significance of *p* < 0.05 was used in all analyses.

## Results

**Fig. 1:**
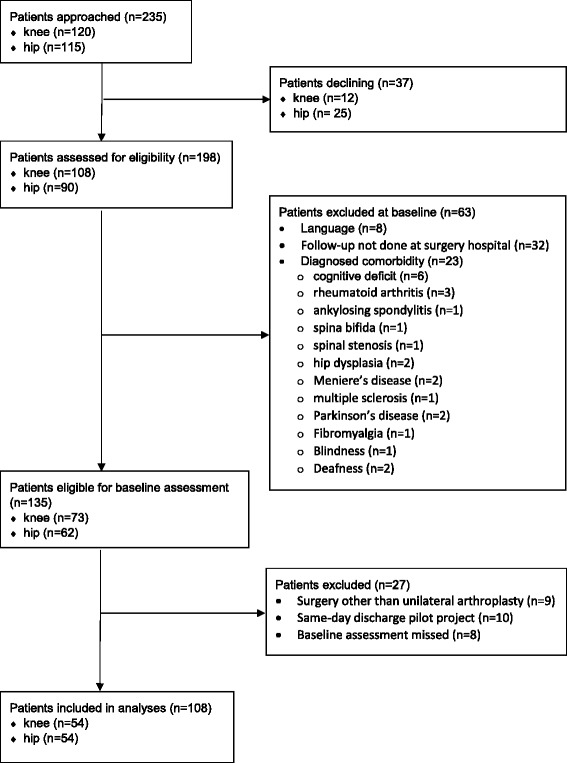
Patient recruitment flow diagram

Figure 1 outlines the recruitment process of patients. There were no significant differences with regards to age or gender when comparing participants to non-participants. Of the 108 participants, 54 were women (50%). The average age was 64 years (SD = 12.5 years), while the average body mass index (BMI) was 30.4 (SD = 6.2). 34 patients (31%) had received a previous hip or knee arthroplasty. The median hospital length of stay was 3 days (SD = 1.7). Table 1 provides descriptive data for the outcome measures. For the TUG, 16 patients (15%) were unable to complete the test on postoperative day 1, while 2 patients (2%) were unable on postoperative day 2. All outcomes showed a deterioration of the condition between preoperative and postoperative day 1 measures, followed by a gradual improvement from postoperative day 1 except for the PQRS emotion and cognition dimensions, and the RHDS support subscale. Knee patients had significantly poorer outcomes when compared to hip patients for ILOA at postoperative day 1, TUG at 6 weeks, RHDS support subscale at day 1 and 2, RHDS global and personal status subscales at day 2, and PQRS pain subscale at week 2 and 6. Although they did not reach significance, almost all other outcomes were poorer for knee patients for all periods.

**Table 1:** Averages with standard deviations of the outcome measures, and percentage of patients recovered according to the PQRS

	Preoperative		Postoperative							
			1-day		2-day		2-week		6-week	
	Hip	Knee	Hip	Knee	Hip	Knee	Hip	Knee	Hip	Knee
Timed-Up-and-Go (sec.)	10.8 (4.6)	11.9 (4.3)	60.7 (49.9)	79.3 (57.9)	40.6 (34.1)	43.2 (26.9)	12.4 (4.3)	13.9 (4.1)	9.4 (3.0)	10.8 (2.6)*
Iowa Level of Assistance Scale (/50)	0.3 (1.0)	0.2 (1.0)	16.7 (9.1)	22.0 (11.6)*	11.1 (7.7)	14.1 (9.0)	3.1 (3.8)	1.0 (1.8)	0.9 (2.8)	0.2 (0.6)
Readiness for Hospital Discharge Scale										
global (/10)	-	-	7.2 (1.4)	6.7 (1.5)	7.9 (1.4)	7.1 (1.6)*	-	-	-	-
personal status (/10)	-	-	5.5 (2.2)	4.8 (2.1)	6.9 (1.7)	5.9 (1.8)*	-	-	-	-
knowledge (/10)	-	-	7.2 (1.8)	7.0 (2.0)	7.8 (1.8)	7.2 (2.1)	-	-	-	-
coping (/10)	-	-	7.6 (2.2)	7.0 (2.2)	7.8 (2.1)	7.3 (2.1)	-	-	-	-
support (/10)	-	-	9.3 (1.1)	8.4 (1.9)*	9.4 (1.0)	8.4 (2.1)*	-	-	-	-
Postoperative quality of recovery Scale (% recovered)										
Pain	-	-	64.8%	47.2%	64.0%	51.0%	88.0%	67.3%*	94.2%	75.5%*
Emotion	-	-	88.9%	83.0%	86.0%	86.3%	92.0%	78.8%	86.5%	81.1%
Function	-	-	0%	3.8%	12.0%	16.3%	40.0%	57.7%	77.6%	86.8%
Cognition	-	-	77.4%	84.9%	74.0%	70.6%	80.4%	90.4%	86.3%	81.1%

*: *p* < 0.05 between hip and knee patients

For floor and ceilings effects, the ILAS demonstrated floor effects preoperatively (90.6% of hip patients having the lowest score; 92.6% of knee patients), 2 weeks (41.2% of hip patients; 68.6% of knee patients) and 6 weeks postoperatively (87.2% of hip patients; 92.2% of knee patients), while the support subscale of the RHDS demonstrated ceiling effects at postoperative day 1 (46.3% of hip patients; 32.1% of knee patients) and day 2 (60.0% of hip patients; 39.2% of knee patients). For the PQRS, more than 85% of patients were deemed recovered on the pain scale at 2 and 6 weeks for hip patients, cognition subscale at 2 weeks for knee patients and 6 weeks for hip patients, function subscale at 6 weeks for knee patients, and on the emotion subscale at all periods for hip patients and day 2 for knee patients.

SRM results and proportion of patients improved on the PQRS are found in Table 2. A large change in the TUG and ILAS from preoperative to postoperative can be seen, followed by larger changes for the ILAS when compared to the TUG in the first two postoperative weeks, but a larger change for the TUG from 2–6 weeks. The personal status subscale of the RHDS showed the largest change of all RHDS subscales, with small to trivial changes for the other subscales. For the PQRS, the pain and function dimensions showed the most improvement, while the emotion and cognition dimensions changed much less.

**Table 2:** Standardized Response Means of the outcome measures, and proportion of improved patients for the Postoperative Quality of Recovery Scale

	Preoperative to postoperative day 1		Postoperative day 1 to day 2		Postoperative day 2 to week 2		Postoperative week 2 to week 6	
	Hip	Knee	Hip	Knee	Hip	Knee	Hip	Knee
Timed-Up-and-Go	1.03	1.17	0.49	0.69	0.85	1.09	1.24	1.02
Iowa Level of Assistance Scale	1.91	1.90	0.80	0.92	1.02	1.44	0.62	0.47
Readiness for Hospital Discharge Scale								
global	-	-	0.66	0.45	-	-	-	-
personal status	-	-	0.85	0.68	-	-	-	-
knowledge	-	-	0.45	0.20	-	-	-	-
coping	-	-	0.16	0.26	-	-	-	-
support	-	-	0.09	0.06	-	-	-	-
Postoperative Quality of Recovery Scale (% improved)								
Pain	-	-	16.0%	20.0%	23.4%	26.5%	10.4%	11.8%
Emotion	-	-	8.0%	14.0%	12.8%	8.2%	6.3%	13.7%
Function	-	-	12.2%	12.8%	31.9%	44.7%	41.3%	31.4%
Cognition	-	-	16.3%	2.0%	16.7%	22.4%	12.5%	3.9%

Table 3 details the areas under the curve MIC results. The changes in the global scale (MIC of 1.1/10; sensitivity 57.1%; specificity: 89.7%) and personal status subscale of the RHDS (MIC of 2.4/10; sensitivity 47.6%; specificity: 96.6%) for knee patients, and the TUG change to week 6 (MIC of 43.2 s; sensitivity 50.0%; specificity: 94.4%) for hip patients were significantly related to patient perceived improvement. The changes in the emotion dimension to day 2 (sensitivity 23.8%; specificity 100%) and to week 2 (sensitivity 8.3%; specificity 93.3%) of the PQRS for hip patients were also significantly related to perceived patient improvement. However, the significance of the emotion dimension change is skewed by the low prevalence of hip patients improving on that dimension (see Tables 1 and 2) and high specificity.

**Table 3:** Minimal important change areas under the curve of the outcome measures, and p values for the PQRS

	Postoperative day 1 to day 2		Postoperative day 1 to week 2		Postoperative day 1 to week 6	
	Hip	Knee	Hip	Knee	Hip	Knee
	Areas under the curve					
Timed-Up-and-Go	0.54 (0.37–0.72) (*p* = .62)	0.50 (0.34–0.67) (*p* = .96)	0.65 (0.48–0.83) (*p* = .10)	0.52 (0.35–0.70) (*p* = .79)	0.68 (0.51–0.85) (*p* = .05)*	0.51 (0.28–0.73) (*p* = .96)
Iowa Level of Assistance Scale	0.63 (0.46–0.80) (*p* = .15)	0.44 (0.29–0.61) (*p* = .54)	0.61 (0.44–0.79) (*p* = .22)	0.42 (0.26–0.58) (*p* = .40)	0.62 (0.45–0.80) (*p* = .18)	0.44 (0.27–0.61) (*p* = .61)
Readiness for Hospital Discharge Scale						
global	0.55 (0.37–0.72) (*p* = .59)	0.68 (0.51–0.84) (*p* = .03)*	-	-	-	-
personal status	0.56 (0.39–0.74) (*p* = .47)	0.71 (0.55–0.87) (*p* = .01)*	-	-	-	-
knowledge	0.51 (0.33–0.68) (*p* = .92)	0.63 (0.47–0.79) (*p* = .12)	-	-	-	-
coping	0.57 (0.39–0.74) (*p* = .46)	0.57 (0.40–0.74) (p = .40)	-	-	-	-
support	0.45 (0.28–0.63) (*p* = .60)	0.50 (0.33–0.66) (*p* = .95)	-	-	-	-
Postoperative Quality of Recovery Scale	Chi-square values					
Pain	*p* = 1.00	*p* = 0.49	*p* = 0.46	*p* = 0.75	*p* = 0.06	*p* = 0.46
Emotion	*p* = 0.05*	*p* = 0.12	*p* = 0.05*	*p* = 1.00	*p* = 0.06	*p* = 0.57
Function	*p* = 1.00	*p* = 0.67	*p* = 0.75	*p* = 0.36	*p* = 0.11	*p* = 0.66
Cognition	*p* = 0.66	*p* = 1.00	*p* = 0.34	*p* = 0.34	*p* = 0.21	*p* = 0.14

*p < 0.05

The relationship results between patient-reported outcome measures and performance measures can be found in Table 4. The RHDS global score and all subscales, except support, were moderately related to performances measures, mostly on postoperative day 2. The personal status subscale was the only subscale significantly related to performance measures on both postoperative days, with generally higher coefficients than the other subscales. For the PQRS, the pain dimension at 2 weeks and function dimension at 6 weeks were significantly related to performance measures in hip patients. As for the relationship between performance measures (TUG and ILAS; not shown in table), the correlation coefficient varied from 0.63–0.85 (p < 0.05) for the periods ranging from preoperative to postoperative day 2, but decreased to 0.36–0.47 (p < 0.05) at week 2 and 0.14–0.54 at week 6.

**Table 4:** Relationship between performance measures and patient-reported outcome measures

	ILAS								TUG							
	POD1		POD2		2wk		6wk		POD1		POD2		2wk		6wk	
	Hip	Knee	Hip	Knee	Hip	Knee	Hip	Knee	Hip	Knee	Hip	Knee	Hip	Knee	Hip	Knee
Readiness for Hospital Discharge Scale	Pearson coefficients															
global	-0.38*	-0.15	-0.53*	-0.34*	-	-	-	-	-0.21	-0.11	-0.64*	-0.39*	-	-	-	-
personal status	-0.50*	-0.27	-0.53*	-0.46*	-	-	-	-	-0.35*	-0.23	-0.61*	-0.35*	-	-	-	-
knowledge	-0.21	-0.06	-0.46*	-0.24*	-	-	-	-	-0.10	-0.02	-0.58*	-0.32*	-	-	-	-
coping	-0.19	0.01	-0.51*	-0.33*	-	-	-	-	-0.05	0.08	-0.63*	-0.42*	-	-	-	-
support	-0.05	-0.09	-0.07	-0.07	-	-	-	-	0.03	-0.09	-0.11	-0.13	-	-	-	-
Postoperative Quality of Recovery Scale	*p*-value of *t*-tests															
Pain	0.18	0.57	0.96	0.74	0.00*	0.33	0.59	0.64	0.35	0.38	0.85	0.27	0.07*	0.98	0.73	0.21
Emotion	0.48	0.15	0.80	0.93	0.96	0.20	0.70	0.40	0.81	0.17	0.40	0.91	0.39	0.59	0.64	0.50
Function	-	0.65	0.51	0.54	0.35	0.25	0.05*	0.47	-	0.81	0.47	0.92	0.09	0.24	0.04*	0.15
Cognition	0.89	0.63	0.71	0.08	0.96	0.55	0.54	0.81	0.86	0.78	0.69	0.52	0.65	0.33	0.10	0.40

*p < 0.05

## Discussion

This study compared the clinimetric properties of four tools to assess the condition of knee or hip arthroplasty patients during the acute recovery phase after surgery. The instruments demonstrated the generally negative impact of surgery on function, on both patient-perceived and actual functional performance. Results also showed that function usually did not return to preoperative values before the 6-week interval. Similar recovery patterns have been demonstrated in other studies [28–31]. Improving function shortly after surgery can help prevent deconditioning, increase patient safety and decrease the need for external resources after hospital discharge. The results of the present study highlight the need of a valid outcome measure to assess function shortly after surgery, in order to evaluate the efficacy of perioperative interventions aimed at improving short-term function. Also, the anticipated impact of surgery on function should be discussed pre and postoperatively with the patient, as it has been shown that patient satisfaction can decrease if patients experience slower recovery than what they expected [32]. When comparing knee with hip arthroplasty, patients undergoing knee arthroplasty generally tended to have poorer outcomes both pre and postoperatively. This is especially the case for pain, where recovery was significantly less for knee compared to hip patients. Pain could explain why poorer function was also obtained for knee patients. Poorer outcomes for knee compared to hip arthroplasty have been demonstrated in other studies [28, 33]. The amount of change in outcomes was however similar for both groups, although recovery (especially for the TUG, ILAS and PQRS function) was more in the first 2 weeks for knee patients, and more in the period from 2 to 6 weeks for hip patients. This could be explained by the poorer preoperative outcomes for knee patients, allowing quicker recovery after surgery to preoperative outcome levels in this patient subgroup.

Although surgery had a considerable impact on function, other dimensions assessed by PROMs such as cognitive, emotional and social factors were much less affected. Postoperatively, function improved considerably while cognitive, emotional and support factors were stable for the majority of patients. These dimensions therefore appear less critical as an outcome measure with patients undergoing arthroplasty during the acute recovery period. Other studies have demonstrated the limited importance of psychosocial dimensions in the acute recovery phase of arthroplasty patients [34, 35]. Arthroplasty is generally planned well in advance, with generally predictable results when compared to other types of surgeries, such as unplanned cardiac surgery. It is therefore possible that arthroplasty is generally less emotionally involving than other types of surgeries, limiting the usefulness of psychosocial outcome measures in the acute recovery phase.

When comparing performance measures with PROMs, results show that the amount of change was similar for both categories of tools in the days after surgery. However, the relationships between performance measures and PROMs were moderate at best. PROMs were related to patient perceived improvement in the days following surgery, whereas performance measures were related to patient perceived improvement only after 6 weeks. It thus appears that patient perception of function and actual functional performance are somewhat different dimensions in the days after surgery, but more related after several weeks. In the first days after surgery, it is probably difficult for patients to determine their level of function since they have performed limited functional tasks. Also, perception of function is probably influenced by other factors present shortly after surgery, such as pain, nausea/dizziness and level of alertness. When the effects of anaesthesia subside and patients gradually increase their levels of activity in the weeks after surgery, it is probably easier for patients to ascertain their level of function. It could therefore be important to assess both perceived function and functional performance in the days after surgery, but not necessarily after 6 weeks. Another factor that could explain this discrepancy could be previous arthroplasties, where perceptions of function for patients having undergone previous arthroplasties could be different from patients undergoing an arthroplasty for the first time, based on past experience and clearer expectations. Other studies have demonstrated the discrepancies between perceived function and functional performance after arthroplasty [3–5, 36, 37]. Studies are however needed to understand these discrepancies and assess their impact, since the consequences of patients over or underestimating their perceived function is not known. When there is a discrepancy, it could be argued that the focus should be functional performance, since it objectively demonstrates what the patient is capable of doing. Conversely, it could also be argued that patient perception is the most important, since it reflects what the patient is going to try at home. A clinical objective after the functional performance assessment could be to educate the patient, in order to reduce this discrepancy. Research is however needed to determine how to manage these discrepancies.

Both performance measures (ILAS and TUG) were highly related in the days after surgery, but less in the weeks after. This reduced relationship could be explained by the ceiling effect of the ILAS in the weeks after surgery. The ILAS changed more in the days after surgery, while the TUG changed more in the weeks after. These results seem to demonstrate that the ILAS assesses more basic function than the TUG, which is consistent with the tasks assessed by both tools. The ILAS assesses the patient’s capacity to do basic tasks in isolation, while the TUG is a timed combination of tasks. Although there were slight result differences between these tools, they were quite similar in the way they performed. The TUG however takes much less time, space and resources than the ILAS. The TUG can also be used over a wider period since it did not show a ceiling effect as the ILAS did. An MIC of 43.4 s over 6 weeks was also obtained for the TUG for hip patients, while none was obtained for the ILAS. Thus, the TUG appears superior to the ILAS to assess function in the first 6 weeks following arthroplasty. Several studies have also demonstrated the validity of TUG in arthroplasty patients, while studies are less frequent for the ILAS [6]. Although specificity was high for the TUG MIC, sensitivity was moderate. Thus, patients below the MIC TUG cut-off could be misclassified as not having improved when, in fact, they did improve. Conversely, patients above the cut-off can be considered improved with more certainty. High specificity combined with moderate sensitivity has been found in another study aimed at identifying the TUG MIC in patients with hip osteoarthritis [21].

Contrary to other studies that demonstrated the lack of responsiveness of PROMs in the acute recovery phase [3–5, 36, 37], the personal status subscale of the RHDS was shown to be responsive according to SRMs. MICs were also obtained for the global and personal status subscale of the RHDS for knee patients. These results are probably attributable to the fact that the RHDS was specifically developed for the postoperative recovery phase, where other PROMs studied in arthroplasty patients were originally developed for osteoarthritis patients and assess more advanced tasks. The RHDS can however only be used during hospitalization, which is generally brief in arthroplasty, limiting its usefulness as an outcome measure. As for the PQRS, it appears more appropriate in the longer term since it assesses the patient’s capacity to return to preoperative status. There thus appears to be a need for a postoperative recovery PROM developed specifically for arthroplasty patients, since there are currently none.

The study results are limited to the context in which they were collected, i.e. a large Canadian university-affiliated hospital performing a significant number of arthroplasties. Care should be taken when transposing the present results to other settings. This study should therefore be replicated in other contexts. The rate of ineligible patients is also relatively high, although this can mostly be explained by out-of-town patients not available for follow-ups. Subgroup analyses comparing knee and hip arthroplasties were also not possible due to the limited sample size. Finally, outcome measure results could have been influenced by the presence of osteoarthritis in other joints, but this was not assessed.

## Conclusion

Out of the four tools assessed, the TUG and RHDS personal status subscale demonstrated the best clinimetric properties in the assessment of function shortly after hip or knee arthroplasty, with regards to floor and ceiling effects, amount of change, relationship with patient perceived improvement and ability to calculate an MIC. The relationship between the TUG and RHDS personal status were moderate, indicating that these are to an extent different dimensions that could be both useful to obtain a complete picture of patient function.

## Abbreviations

ILAS: Iowa Level of Assistance Scale
MIC: minimal important change
PQRS: Postoperative Quality of Recovery Scale
PROMs: patient-reported outcome measures
RHDS: Readiness for Hospital Discharge Scale
ROC: Receiver Operating Characteristics
SRM: standardised response mean
TUG: Timed Up and Go
